# Supplementary material for: Evidence for loss and reacquisition of alcoholic fermentation in a fructophilic yeast lineage
Source: eLife. 2018 Apr 12;7:e33034. doi: 10.7554/eLife.33034 (PMC5897096; doi:10.7554/eLife.33034)
Supplement: Figure 2—source data 1. [file elife-33034-fig2-data1.docx]

**Figure 2- source data 1**

| **Species** | **Abb.** | **Strain(s)** | **Genome**  **database** | **Rpa1** | **Rpa2** | **Rpb1** | **Rpb2** | **Rpc1** | **Rpc2** |
| --- | --- | --- | --- | --- | --- | --- | --- | --- | --- |
| *Aspergillus niger* | *A.* niger | CBS 513.88 | BROAD/JGI | EHA18239.1 | CAK45911.1 | EHA27022.1 | XP_001395161.2 | XP_001393726.1 | XP_001388998.2 |
| *Fusarium graminearum (Gibberella zeae)* | *F. graminearum* | PH-1 | SGD/BROAD | XP_384467.1 | XP_385803.1 | XP_381092.1 | FGSG_02659.3 | XP_390036.1 | XP_381019.1 |
| *Blastobotrys adeninivorans* | *B. adeninivorans* |  | NCBI | ARAD1A03894g | ARAD1D42196g | ARAD1C32846g | ARAD1D00352g | ARAD1C14146g | ARAD1B19536g |
| *Ascoidea rubescens* | *A. rubescens* | NRRL Y17699 | JGI | 158172 | 121398 | 152893 | 74872 | 37761 | 73423 |
| *Babjeviella inositovora* | *A. inositovora* | NRRL Y-12698 | JGI | 163242 | 159233 | 36081 | 159736 | 65579 | 160834 |
| *Candida tenuis* | *C. tenuis* | NRRL Y-1498 | JGI | EGV63759.1 | EGV64922.1 | 122800^a^ | EGV66444.1 | EGV61865.1 | EGV61757.1 |
| *Candida albicans* | *C. albicans* | WO-1 | SGD/BROAD | XP_710761.1 | XP_720354.1 | EEQ44066.1 | XP_718439.1 | EEQ44828.1 | XP_710813.1 |
| *Candida apicola* | *C. apicola* | NRLL Y-50540 | NCBI | LBNK01000021.1 | LBNK01000009.1 | LBNK01000005.1 | LBNK01000006.1 | LBNK01000007.1 | LBNK01000009.1 |
| *Candida arabinofermentans* | *C.. arabinofermentans* | NRRL YB-2248 | JGI | 237815 | 196656 | 28373 | 97166 | 5073 | 236521 |
| *Candida caseinolytica* | *C. caseinolytica* | NRRL Y-17796 | JGI | 103917 | 71761 | 65867^a^ | 32658 | 57439^a^ | 76153 |
| *Candida dubliniensis* | *C. dubliniensis* | CD36 | SGD/YGOB | XP_002417643.1 | XP_002421229.1 | XP_002422523.1 | XP_002416823.1 | XP_002420293.1 | XP_002422092.1 |
| ***Candida galacta**** | *C. galacta* | yHMPu5000041860 | Local database | Scaffold7 | Scaffold13 | Scaffold30 | Scaffold69 | Scaffold15 | Scaffold19 |
| *Candida glabrata* | *C. glabrata* | CBS 138 | SGD/YGOB | XP_445928.1 | XP_447785.1 | XP_447415.1 | XP_448959.1 | XP_449275.1 | XP_448895.1 |
| *Candida magnoliae* | *C. magnoliae* | PYCC 2903 | NCBI | ALK02047.1 | ALK02048.1 | ALK02049.1 | ALK02050.1 | ALK02051.1 | ALK02052.1 |
| *Candida parapsilosis* | *C. parapsilosis* | CDC317 | BROAD/NCBI | CCE41808.1 | CCE41117.1 | CCE42368.1 | CCE40898.1 | CCE40988.1 | CCE42811.1 |
| *Candida tanzawaensis* | *C. tanzawaensis* | NRRL Y-17324 | JGI | 27643 | 51620 | 205015 | 315886 | 20117 | 25811 |
| *Candida tropicalis* | *C. tropicalis* | MYA-3404 | SGD/BROAD | XP_002548986.1 | XP_002550740.1 | Supercontig10^d^ | XP_002550158.1 | XP_002546294.1^d^ | XP_002545804.1 |
| *Candida versatilis* | *C. versatilis* | JCM 5958 | RIKEN | 00909 | 01065 | 03613 | 01450 | 03737 | 00838 |
| *Clavispora lusitaniae* | *C. lusitaniae* | ATCC 42720 | BROAD/NCBI | XP_002614662.1 | XP_002617668.1 | XP_002616227.1 | CLUG_03629.1^a^ | XP_002617549.1 | XP_002617014.1 |
| *Cyberlindnera jadinii* | *C. jadinii* | NBRC 0988 | NCBI | BAEL01000045.1 | BAEL01000080.1 | BAEL01000145.1 | BAEL01000002.1^d^ | BAEL01000017.1 | BAEL01000068.1 |
| *Debaryomyces hansenii* | *D. hansenii* | CBS767 | SGD/NCBI | XP_002770194.1 | XP_461338.1 | XP_002770038.1 | XP_002770594.1 | XP_462102.2 | XP_002777730.1 |
| *Dekkera bruxellensis* | *D. bruxellensis* | AWRI1499 | NCBI | 68261 | EIF46931.1 | EIF46824.1 | 7243 | EIF47620.1 | 87139 |
| *Eremothecium cymbalariae* | *E. cymbalariae* | DBVPG#7215 | NCBI/YGOB | Chr 5 | XP_003644970.1 | XP_003648178.1 | XP_003646391.1 | XP_003647083.1 | XP_003648395.1 |
| *Eremothecium gossypii* | *E. gossypii* | ATCC 10895 | SGD/NCBI | NP_984470.2 | NP_982975.2 | NP_984182.2 | NP_985951.1 | NP_985109.2 | NP_983821.2 |
| *Hyphopichia burtonii* | *H. burtonii* | NRRL Y-1933 | JGI | 235620 | 10853 | 213139 | 152096 | 203469 | 105789 |
| *Kazachstania africana* | *K. africana* | CBS 2517 | YGOB | CCF60409.1 | CCF57324.1 | CCF56404.1 | CCF58398.1 | CCF58150.1 | CCF59234.1 |
| *Kazachstania naganishii* | *K. naganishii* | CBS 8797 | YGOB | Chr 4 | Chr 4 | Chr 3 | Chr 2 | Chr 2 | Chr 1 |
| *Kluyveromyces aestuarii* | *K. aestuarii* | ATCC 18862 | NCBI | AEAS01000160.1 | AEAS01000155.1 | AEAS01000088.1 | AEAS01000100.1 | AEAS01000212.1 | AEAS01000006.1 |
| *Kluyveromyces lactis* | *K. lactis* | NRRL Y-1140 | SGD/YGOB | XP_454356 | XP_456115.1 | XP_451816.1 | XP_455310.1 | XP_451784.1 | XP_454912.1 |
| *Kluyveromyces wickerhamii* | *K. wickerhamii* | UCD 54-210 | NCBI | AEAV01000083.1 | AEAV01000033.1 | AEAV01000150.1 | AEAV01000240.1 | AEAV01000216.1 | AEAV01000246.1 |
| *Komagataella pastoris* | *K. pastoris* | GS115 + CBS 7435 | SGD/NCBI | XP_002491799.1 | XP_002490446.1 | XP_002492795.1 | XP_002491011.1 | CCA40852.1 | XP_002493647.1 |
| *Lachancea kluyveri* | *L. kluyveri* | NRRL Y-12651 | SGD/YGOB | Chr 2 | Chr 2 | Chr 6 | Chr 7 | Chr 7 | Chr 8 |
| *Lachancea thermotolerans* | *L. thermotolerans* | CBS 6340 | SGD/YGOB | XP_002552795.1 | XP_002555777.1 | XP_002555968.1 | XP_002555168.1 | XP_002554891.1 | XP_002553136.1 |
| *Lachancea waltii* | *L. waltii* | NCYC 2644 | SGD/YGOB | kwal_s_26 | kwal_s_14 | kwal_s_56 | kwal_s_47 | kwal_s_55 | kwal_s_26 |
| *Lipomyces starkeyi* | *L. starkeyi* | NRRL Y-11557 | JGI | 336068 | 4553 | 75854 | 73345 | 1849 | 331 |
| *Lodderomyces elongisporus* | *L. elongisporus* | NRRL YB-4239 | BROAD/NCBI | XP_001527964.1 | XP_001523265.1 | XP_001523387.1 | XP_001526602.1 | XP_001523103.1 | XP_001525350.1 |
| *Metschnikowia bicuspidata* | *M. bicuspidata* | NRRL YB-4993 | JGI | 101699 | 30063 | 170566 | 32161 | 12577 | 32049 |
| *Meyerozyma guilliermondii* | *M. guilliermondii* | ATCC 6260 | BROAD/NCBI | EDK39611.2 | EDK36311.2^a,d^ | XP_001487729.1 | EDK38735.2 | EDK40245.2 | EDK39998.2 |
| *Millerozyma farinosa* | *M. farinosa* | CBS 7064 | SGD/NCBI | CCE81062.1 | CCE86373.1 | CCE87208.1 | CCE78311.1 | CCE88859.1 | CCE82943.1 |
| *Nadsonia fulvescens var. elongata* | *N. fulvescens* | DSM 6958 | JGI | 49068 | 50775 | 44819 | 82421 | 70621 | 53794 |
| *Naumovozyma castellii* | *N. castellii* | CBS 4309 | YGOB | XP_003676913.1 | XP_003675778.1 | XP_003674328.1 | XP_003677897.1 | XP_003677861.1 | XP_003674628.1 |
| *Ogataea polymorpha* | *O. polymorpha* | NCYC 495 leu1.1 | JGI | 64765 | 28517 | 62103 | 64873 | 17249^a^ | 64464 |
| *Ogataea parapolymorpha* | *O. parapolymorpha* | DL-1 | NCBI | EFW96323.1 | EFW95149.1 | EFW94818.1 | EFW96371.1 | EFW95798.1 | EFW95940.1 |
| *Pachysolen tannophilus* | *P. tannophilus* | NRRL Y-2460 | JGI/NCBI | CAHV01000103.1 | CAHV01000123.1 | CAHV01000062.1 | CAHV01000236.1^d^ | CAHV01000062.1 | CAHV01000160.1 |
| *Pichia kudriavzevii* | *P. kudriavzevii* | M12 | NCBI | ALNQ01000183.1 | ALNQ01000058.1 | ALNQ01000167.1 | ALNQ01000064.1 | ALNQ01000075.1 | ALNQ01000054.1 |
| *Pichia membranifaciens* | *P. membranifaciens* | NRRL Y-2026 | JGI | 15631 | 15732 | 28620 | 71110 | 73178 | 15919 |
| *Saccharomyces cerevisiae* | *S. cerevisiae* | S288c | SGD/BROAD | P10964.2 | P22138.1 | P04050.2 | P08518.2 | P04051.1 | P22276.2 |
| *Saccharomyces eubayanus* | *S. eubayanus* | FM1318 | NCBI | Contig0673 | Contig0661 | Contig0455  Contig0592^b^ | Contig587 | Contig620 | Contig583^d^ |
| *Saccharomyces kudriavzevii* | *S. kudriavzevii* | IFO 1802 | SSS Website | Scaffold8 | Scaffold34 | Scaffold3 | Scaffold58 | Scaffold51 | Scaffold8 |
| *Saccharomyces mikatae* | *S. mikatae* | IFO 1815 | SSS Website  SGD/BROAD | Scaffold13 | Scaffold1 | Scaffold8 | Scaffold32 | Scaffold32 | Scaffold13 |
| *Saccharomyces paradoxus* | *S. paradoxus* | NRRLY-17217 | SSS Website  NCBI | Scaffold15 | Scaffold16 | AABY01000121.1^d^ | Scaffold15 | AABY01000093.1^d^ | Scaffold15 |
| *Saccharomyces uvarum* | *S. uvarum* | CBS 7001 | SSS Website  SGD/BROAD | Scaffold8 | Scaffold2 | Scaffold11 | Scaffold8 | Scaffold16 | Scaffold8 |
| *Saprochaete clavata* | *Sa. clavata* | CNRMA 12.647 | NCBI | CBXB010000009.1 | CBXB010000051.1 | CBXB010000080.1 | CBXB010000096.1 | CBXB010000062.1 | CBXB010000058.1 |
| *Scheffersomyces stipitis* | *S. stipitis* | CBS 6054 | JGI/NCBI | XP_001386054.2 | XP_001384965.2 | XP_001386996.2 | XP_001387366.2 | XP_001387687.2 | XP_001383024.1 |
| *Spathaspora passalidarum* | *S. passalidarum* | NRRL Y-27907 | JGI/NCBI | EGW32330.1 | EGW30093.1 | EGW33664.1 | EGW34678.1 | EGW35786.1 | EGW34142.1 |
| *Starmerella bacillaris* | *St. bacillaris* | PYCC 3044 | NCBI | AGQ04600.1 | AGQ04601.1 | AGQ04602.1 | AGQ04603.1 | AGQ04604.1 | AGQ04605.1 |
| *Starmerella bombicola* | *St. bombicola* | PYCC 5882 | NCBI | ALK02053.1 | ALK02054.1 | ALK02055.1 | ALK02056.1 | ALK02057.1 | ALK02058.1 |
| *Sugiyamaella lignohabitans* | *Su. lignohabitans* | NRRL YB-1473 | NCBI | ANB14923.1 | ANB15930.1 | ANB15624.1 | ANB15047.1 | ANB12611.1 | ANB12980.1 |
| *Tetrapisispora blattae* | *T. blattae* | CBS 6284 | YGOB | CCH61247.1 | CCH61446.1 | CCH61759.1 | CCH58045.1 | CCH58170.1 | CCH59253.1 |
| *Tetrapisispora phaffii* | *T. phafii* | CBS 4417 | YGOB | XP_003686917.1 | XP_003686698.1 | XP_003683819.1 | XP_003687546.1 | XP_003686816.1 | XP_003683621.1 |
| *Torulaspora delbrueckii* | *T. delbrueckii* | CBS 1146 | YGOB | XP_003683470.1 | XP_003679887.1 | XP_003680332.1 | XP_003678904.1 | XP_003681653.1 | XP_003679108.1 |
| *Trichomonascus petasosporus* | *T. petasosporus* | NRRL YB-2093 | JGI | 161286 | 157270 | 156742 | 153131 | 160262 | 131780 |
| *Vanderwaltozyma polyspora* | *V. polyspora* | DSM 70294 | YGOB/NCBI | XP_001643648.1 | XP_001645652.1 | XP_001642173.1 | XP_001642238.1 | XP_001643927.1 | XP_001643385.1 |
| *Wickerhamiella domercqiae* | *W. domercqiae* | PYCC 3067 | NCBI | ALK02059.1 | ALK02060.1 | ALK02061.1 | ALK02062.1 | ALK02063.1 | ALK02064.1 |
| *Wickerhamomyces anomalus* | *W. anomalus* | NRRL Y-366 | JGI | 62320 | 99340 | 60320 | 53842 | 31590 | 36757^a^ |
| *Yarrowia lipolytica* | *Y. lipolytica* | CLIB122 | SGD/NCBI | XP_505388.1 | XP_503752.1 | XP_501909.2 | XP_502376.1 | XP_502142.1 | XP_500966.1 |
| *Zygosaccharomyces bailii* | *Zygbai* | CLIB 213 | NCBI | [CDF90606.1](http://www.ncbi.nlm.nih.gov/protein/523424266?report=genbank&log$=prottop&blast_rank=2&RID=GHAVDSMJ016) | [CDF90198.1](http://www.ncbi.nlm.nih.gov/protein/523423876?report=genbank&log$=prottop&blast_rank=1&RID=GH0JHPF501R) | [CDF90990.1](http://www.ncbi.nlm.nih.gov/protein/523424637?report=genbank&log$=prottop&blast_rank=1&RID=GHAURXBP01R) | [CDF89910.1](http://www.ncbi.nlm.nih.gov/protein/523423602?report=genbank&log$=prottop&blast_rank=3&RID=GHAZ5CDM014) | [CDF90706.1](http://www.ncbi.nlm.nih.gov/protein/523424361?report=genbank&log$=prottop&blast_rank=2&RID=GHAYFWYK016) |  |
| *Zygosaccharomyces kombuchaensis* | *Zygkom* | CBS 8849 | Local database | scaffold00837 | scaffold00371 | scaffold00560 | scaffold00711 | scaffold00297 | scaffold00837 |
| *Zygosaccharomyces rouxii* | *Zygrou* | CBS 732 | SGD/JGI | XP_002496630.1 | XP_002499065.1 | XP_002497454.1 | XP_002496945.1 | XP_002497625.1 | XP_002497679.1 |

**Figure 2- source data 1- Complete list of fungal taxa, abbreviated species names, genome databases and accession numbers of RNA polymerase proteins for the species used to construct the species tree (Figure 2A and Figure 8)**. Abbreviated species names (Abb.) are given for each species as used in the phylogeny. For sequences retrieved from JGI databases, the Protein ID number is given. ^a^original protein prediction was modified to correct error. ^b^sequence spanned multiple contigs. ^c^partial sequence not used. ^d^partial sequence. W/S-clade species are highlighted in blue. Species used as outgroups are highlighted in magenta.For JCM strains, draft genome data was obtained from RIKEN BioResource Center and RIKEN Center for Life Science Technologies through the Genome Information Upgrading Program of the National Bio-Resource Project of the MEXT. *C. galacta* (*****) was only included in the species phylogeny shown in Figure 8.
