# Supplementary material for: Evidence for loss and reacquisition of alcoholic fermentation in a fructophilic yeast lineage
Source: eLife. 2018 Apr 12;7:e33034. doi: 10.7554/eLife.33034 (PMC5897096; doi:10.7554/eLife.33034)
Supplement: Figure 7—source data 2. [file elife-33034-fig7-data2.docx]

**Figure 7-source data 2- Primers for construction of *St. bombicola* deletion mutants.**

| **Mutants (Genotypes)** | **Primers** | **Sequence 5’-3’** | **Description** |
| --- | --- | --- | --- |
| *adh1*∆  (*adh1*∆::*HYG)* | ADH1up_BglII_Fw | CATGTTAGATCTTGACCTTCGCCTTGCT | Amplification of *ADH1* gene plus ~1Kb upstream and downstream |
|  | ADH1down_NotI_Rv | AACATGGCGGCCGCGTTCTGCGTCAGCAAG |  |
|  | GPD_SalI_Fw | TTCAGAGTCGACTCAAGCACTGCGGCAC | Amplification of GPD-HYG-CYC cassette for *ADH1* interruption with SalI and KpnI |
|  | CYC_KpnI_Rv | CAGATAGGTACCGTACCGGCCGCAAATTA |  |
|  | ADH1ko_up_Fw | TGCTTAATCAGCTGAGC | Up and downstream confirmation of correct *ADH1* knock out (KO) cassette integration |
|  | ADH1ko_down _Rv | GATCACCTGCTATTGTC |  |
|  | ADH1excised_Fw | GCTACCTATGCATTCAAC | Primer hybridizes with *ADH1* CDS, to confirm the absence of *ADH1* in the deletion mutant |
| *adh6a*∆ (*adh6a*∆::*HYG)* | Sb_ADH6Aup_SacI_Fw | GACTTAGAGCTCTCAGAATACGCTCACAGAC | Amplification of 1Kb upstream sequence from *ADH6*a |
|  | Sb_ ADH6Adown_SpeI_Rv | TAAGTCACTAGTCATGGTGGATTTCGACG |  |
|  | Sb_ADH6Adown_SmaI_Fw | GACTTACCCGGGCACCCTCAACCTGTGG | Amplification of 1Kb downstream sequence from *ADH6a* |
|  | Sb_ ADH6Adown_SalI_Rv | TGCTAAGTCGACACTATTCCGTGCATGCTG |  |
|  | SbADH6a_KOconf_Fw | TGCTTTCATCTCCGAGC | Upstream and downstream confirmation of correct integration of *ADH6a* deletion cassette (by sequencing) |
|  | SbADH6a_KOconf_Rv | CAGGCCTTGAGCAGAC |  |
|  | Sb_ADH6_Fw | ACTATTCCGTGCATGCTG | Amplification of *ADH6a* KO cassette |
|  | Sb_ ADH6_Rv | TCAGAATACGCTCACAGAC |  |
| *adh6b*∆  *(adh6b*∆::*HYG)* | Stabom_ ADH6Bup_SacI_Fw | TAACTAGAGCTCTGTCATGAGCACCGAGC | Amplification of 1Kb upstream sequence from *ADH6b* |
|  | Stabom_ADH6Bup_SpeI_Rv | TAGTTAACTAGTCTGTAAAGGTCATTGTAAATTC |  |
|  | Stabom_ADH6Bdown_SmaI_Fw | TAACTACCCGGGCTTGAGTAATTTATTCGGACG | Amplification of 1Kb downstream sequence from *ADH6b* |
|  | Stabom_ADH6Bdown_SalI_Rv | TAGTTAGTCGACCTCTGCCAGCTTCAGC |  |
|  | SbADH6b_KOconf_Fw | CTGTATGGCTCTCAAAGG | Upstream and downstream confirmation of *ADH6b* KO cassette correct integration (by sequencing) |
|  | SbADH6b_KOconf_Rv | TGGCTAATTGCATTGAGC |  |
|  | SbADH6b_up_Fw | TGTCATGAGCACCGAGC | Amplification of *ADH6b* KO cassette |
|  | SbADH6b_down_Rv | CTCTGCCAGCTTCAGC |  |
| *ffz1*∆  (*ffz1*∆::*HYG*) | SbFFZup_ClaI_Fw | TAACGAATGATCCATTCATGTCAGACTTGC | Amplification of *FFZ1* gene plus ~1Kb upstream and downstream |
|  | Sb_FFZdown_NotI_Rv | TTCGTTAGCGGCCGCATACCTGCCAGATCCG |  |
|  | GPD_FFZup_Fw* | GAAGAGCAACGTCCTTTACAGAAGGTCGAGGTCGACTCAAGCACTGCGGCAC | Hygromycin cassette amplification. *FFZ1* gene was disrupted by transformation of plasmid harbouring the abovementioned fragment along with the Hyg-cassette fragment (obtained using this primer pair) in *S. cerevisiae* as ligation in *E. coli* was unsuccessful. |
|  | CYC_FFZdown_Rv* | TAAAGAAATGTTCTTCAGCGCTCGATCAGAAGATCTGTACCGGCCGCAAATTA |  |
|  | FFZ_up _Fw | TGGTATGCATCTCCAATGT | Amplification of *FFZ1* KO cassette for *St. bombicola* transformation |
|  | FFZ_down Rv | GTCAAAGGTGATGAGCTC |  |
|  | SbFFZ_conf_Fw | GCTACATGTAATCAAACCG | Upstream and downstream confirmation of correct integration of *FFZ1* deletion cassette (by sequencing) |
|  | SbFFZ_conf_Rv | TGGCTAATTGCATTGAGC |  |
|  | FFZ_CDS_Fw | ARGAYCCBTWYAACTG | Primer hybridizes with *FFZ1* CDS, to confirm the absence of *FFZ1* in the deletion mutant |
| *suc2*∆ (*suc2*∆::*HYG*) | SbSUC2up_NotI_Fw | ATCTTCGCGGCCGCTGCTGATGCTGCCGAT | Amplification of *SUC2* gene plus ~1Kb upstream and downstream |
|  | SbSUC2_ClaI_Rv | GAAGATATCGATGTCATTAATAAACAGCCACC |  |
|  | SbSUC2up_Fw | TCTCCATAGATCATGCTTG | Amplification of *SUC2* deletion cassette for *St. bombicola* transformation |
|  | SbSUC2down_Rv | CCATCAGATATGGCTATC |  |
|  | SbSUC2conf_Fw | GAGCTGTCAGTCACAG | Upstream and downstream confirmation of correct integration of *SUC2* deletion cassette (by sequencing) |
|  | SbSUC2conf_Rv | CTTAGCACTAACGAGTAC |  |
|  | SbSUC2_CDS_Fw | AACCTCTAAGTCGCGAG | Primer hybridizes with *SUC2* CDS, to confirm the absence of *SUC2* in the deletion mutant |
| HYG-resistance cassette | GPD_SacI_Sbomb_Fw | CTAGTAGAGCTCTCAAGCACTGCGGCA | Amplification of GPD promoter of *St. bombicola*. SacI and HindIII restriction sites were added to the flanking regions to subsequent ligation to the GPD fragment. |
|  | GPD_HindIII_Sbomb_Rv | TACTAGAAGCTTGAGCTTGACCGGTTCGAAC |  |
|  | Hyg_HindIII_Fw | CTTGATAAGCTTATGAAAAAGCCTGAAC | Amplification of *hygB* gene from pBlueScript-HYG. HindIII and XhoI restriction sites were added to the flanking regions. |
|  | Hyg_XhoI_Rv | GTAATCCTCGAGCTATTCCTTTGCCCT |  |
| *aro10*∆ (*aro10*∆::*HYG*) | SbARO10_ClaI_Fw | ATCTTAATCGATGACAGATAGCTTAACCGA | Amplification of *ARO10* gene plus ~1Kb upstream and downstream |
|  | SbARO10_NotI_Rv | TAAGATGCGGCCGCGCAGCAGCTATTCATGG |  |
|  | SbARO10_Fw | GACAGATAGCTTAACCGA | Amplification of *ARO10* deletion cassette for *St. bombicola* transformation |
|  | SbARO10_Rv | CGCAGCAGCTATTCATGG |  |
|  | SbARO10_conf_Fw | ACAGATAGCTTAACCGA | Upstream and downstream confirmation of correct integration of *ARO10* deletion cassette (by sequencing) |

*Cassette integration step failed in *E.coli* DH5ɑ. Integration was performed in *S. cerevisiae* BY4741 by heterologous recombination as in Goncalves C., et al. 2016.
