# Supplementary material for: Evidence for loss and reacquisition of alcoholic fermentation in a fructophilic yeast lineage
Source: eLife. 2018 Apr 12;7:e33034. doi: 10.7554/eLife.33034 (PMC5897096; doi:10.7554/eLife.33034)
Supplement: Transparent reporting form [file elife-33034-transrepform.docx]

***eLife’s* transparent reporting form**

We encourage authors to provide detailed information *within their submission* to facilitate the interpretation and replication of experiments. Authors can upload supporting documentation to indicate the use of appropriate reporting guidelines for health-related research (see [EQUATOR Network](http://www.equator-network.org/%20)), life science research (see the [BioSharing Information Resource](https://biosharing.org/)), or the [ARRIVE guidelines](http://www.plosbiology.org/article/info:doi/10.1371/journal.pbio.1000412) for reporting work involving animal research. Where applicable, authors should refer to any relevant reporting standards documents in this form.

If you have any questions, please consult our Journal Policies and/or contact us: [editorial@elifesciences.org](mailto:editorial@elifesciences.org).

**Sample-size estimation**

- You should state whether an appropriate sample size was computed when the study was being designed
- You should state the statistical method of sample size computation and any required assumptions
- If no explicit power analysis was used, you should describe how you decided what sample (replicate) size (number) to use

Please outline where this information can be found within the submission (e.g., sections or figure legends), or explain why this information doesn’t apply to your submission:

No explicit power analyses were performed to determine sample sizes or the number of replicates.

**Replicates**

- You should report how often each experiment was performed
- You should include a definition of biological versus technical replication
- The data obtained should be provided and sufficient information should be provided to indicate the number of independent biological and/or technical replicates
- If you encountered any outliers, you should describe how these were handled
- Criteria for exclusion/inclusion of data should be clearly stated
- High-throughput sequence data should be uploaded before submission, with a private link for reviewers provided (these are available from both GEO and ArrayExpress)

Please outline where this information can be found within the submission (e.g., sections or figure legends), or explain why this information doesn’t apply to your submission:

Phenotypic analyses of the mutants were performed using two biological replicates (two different transformants). Two independent experiments were performed with each transformant on separate days. This information was included in the Methods section under the heading “Sugar assimilation, consumption, and metabolite production assessments” and in the legends of Figures 1 and 7.

Private links for phylogeny newick files are indicated in the Methods section under the heading “Phylogenetic analyses of Pdc1, Adh1, Adh6, Suc2 and topology tests“

Genome assemblies were submitted to NCBI and accession numbers are indicated in the methods section under heading “Identification of ADH1, PDC1, ADH6, and SUC2 genes in the W/S clade and related species”; since data is not yet available, assemblies can also be accessed at https://figshare.com/s/905b12b0ac9bf7c46b8f.

**Statistical reporting**

- Statistical analysis methods should be described and justified
- Raw data should be presented in figures whenever informative to do so (typically when N per group is less than 10)
- For each experiment, you should identify the statistical tests used, exact values of N, definitions of center, methods of multiple test correction, and dispersion and precision measures (e.g., mean, median, SD, SEM, confidence intervals; and, for the major substantive results, a measure of effect size (e.g., Pearson's r, Cohen's d)
- Report exact p-values wherever possible alongside the summary statistics and 95% confidence intervals. These should be reported for all key questions and not only when the p-value is less than 0.05.

Please outline where this information can be found within the submission (e.g., sections or figure legends), or explain why this information doesn’t apply to your submission:

Exact P values for topology tests were reported in the text, namely in the sections “HGT and gene duplication of alcohol dehydrogenase genes in W/S-clade species” and “Horizontally transferred SUC2 is essential for sucrose assimilation in St. bombicola”. Exact P-values for phenotypic analyses of the adh and aro10 deletion mutants are given in Figure 7-source data 1.

(For large datasets, or papers with a very large number of statistical tests, you may upload a single table file with tests, Ns, etc., with reference to sections in the manuscript.)

**Group allocation**

- Indicate how samples were allocated into experimental groups (in the case of clinical studies, please specify allocation to treatment method); if randomization was used, please also state if restricted randomization was applied
- Indicate if masking was used during group allocation, data collection and/or data analysis

Please outline where this information can be found within the submission (e.g., sections or figure legends), or explain why this information doesn’t apply to your submission:

Not applicable.

**Additional data files (“source data”)**

- We encourage you to upload relevant additional data files, such as numerical data that are represented as a graph in a figure, or as a summary table
- Where provided, these should be in the most useful format, and they can be uploaded as “Source data” files linked to a main figure or table
- Include model definition files including the full list of parameters used
- Include code used for data analysis (e.g., R, MatLab)
- Avoid stating that data files are “available upon request”

Please indicate the figures or tables for which source data files have been provided:

The numerical data related to Figures 1 and 7 were included in files named respectively

Figure 1-source data 1 and Figure 7-source data 1.

Complete data concerning the Alien index analysis is reported in Figure 2- source data 2.

Accession numbers of proteins used to construct the species phylogenies are reported in Figure 2- source data 1.
